# Anti-Müllerian hormone as a diagnostic marker for testicular degeneration in dogs: insights from cryptorchid models

**DOI:** 10.3389/fvets.2024.1481248

**Published:** 2024-10-07

**Authors:** Florin Petrișor Posastiuc, Guilherme Rizzoto, Nicolae Tiberiu Constantin, George Nicolae, Koen Chiers, Alexandru Ilie Diaconescu, Andreea Iren Șerban, Ann Van Soom, Mario Darius Codreanu

**Affiliations:** ^1^Department of Internal Medicine, Reproduction and Population Medicine, Faculty of Veterinary Medicine, Ghent University, Merelbeke, Belgium; ^2^Department of Clinical Sciences II, Faculty of Veterinary Medicine, University of Agronomic Sciences and Veterinary Medicine of Bucharest, Bucharest, Romania; ^3^Department of Paraclinical Sciences, Faculty of Veterinary Medicine, University of Agronomic Sciences and Veterinary Medicine of Bucharest, Bucharest, Romania; ^4^Department of Pathobiology, Pharmacology and Zoological Medicine, Faculty of Veterinary Medicine, Ghent University, Merelbeke, Belgium; ^5^Department of Preclinical Science, Faculty of Veterinary Medicine, University of Agronomic Sciences and Veterinary Medicine of Bucharest, Bucharest, Romania; ^6^Department of Biochemistry and Molecular Biology, Faculty of Biology, University of Bucharest, Bucharest, Romania

**Keywords:** anti-Müllerian hormone, testicular degeneration, cryptorchidism, spermatogenesis, canine infertility

## Abstract

**Introduction:**

The increasing prevalence of infertility in male dogs in clinical practice mirrors current trends seen in human medicine. Acquired infertility is notably more common in dogs compared to congenital causes, with conditions such as testicular degeneration leading to irreversible loss of fertility. Current diagnostic methods for testicular degeneration, such as histopathological and cytological examinations, rely on testicular biopsy or fine needle aspiration, making them less feasible for routine use. Anti-Müllerian hormone (AMH), produced by Sertoli cells, has emerged as a potential alternative biomarker for testicular health, which can be measured in serum. This study evaluates AMH as a potential marker for testicular degeneration, using cryptorchid dogs as models for impaired fertility and altered testicular histology.

**Methods:**

The relationship between serum AMH levels and AMH tissue expression with impaired spermatogenesis and altered histology was investigated. Serum AMH levels were determined in intact, cryptorchid, and castrated individuals using an immuno-enzymatic ELISA kit and compared between subgroups based on testicular location. Tissue AMH immuno-expression was differentially quantified in two regions of interest (ROIs), the interstitial space and the seminiferous tubule, in both descended and retained gonads. Furthermore, testicles were analyzed using histomorphometric analysis in seminiferous tubules, while spermatogenesis was evaluated using the Johnsen score.

**Results:**

Serum AMH levels were positively correlated with AMH expression assessed in both interstitial space (*ρ* = 0.494, *p* ≤ 0.01) and seminiferous tubules (*ρ* = 0.610, *p* ≤ 0.001). Conversely, serum AMH levels showed a negative correlation with the seminiferous tubule area (*ρ* = −0.435, *p* ≤ 0.05). Smaller seminiferous tubule areas were linked to increased AMH reactivity in both seminiferous tubules (*ρ* = −0.774, *p* ≤ 0.001) and interstitial space (*ρ* = −0.725, *p* ≤ 0.001). Additionally, lower Johnsen scores were associated with higher serum AMH levels (*ρ* = −0.537, *p* ≤ 0.01) and elevated AMH expression in both seminiferous tubules (*ρ* = −0.756, *p* ≤ 0.001) and interstitial space (*ρ* = −0.679, *p* ≤ 0.001).

**Discussion:**

Our results suggest that higher serum levels and tissue expression of AMH are linked to smaller seminiferous tubules and poorer Johnsen scores, reflecting degenerative changes and Sertoli cell dysfunction in retained testicles. Given the similarities in the mechanisms that increase AMH levels in both cryptorchid and non-cryptorchid testicles affected by testicular degeneration, this study recommends using AMH as a marker for diagnosing testicular degeneration in dogs.

## 1 Introduction

Infertility in male dogs is defined as either the inability to mate or the failure to fertilize multiple fertile bitches despite optimal breeding timing (1). This issue is becoming increasingly significant in clinical practice, mirroring the trends seen in human medicine (2, 3). Consequently, there is a growing focus on developing objective predictors for fertility and testicular health in male dogs. Such advancements could facilitate early detection of declining fertility, which would enable timely intervention. Infertility in male dogs is described as either congenital or acquired, with the latter being the more frequently observed cause of reproductive failure (4). Acquired infertility, linked to factors such as hormonal disturbances, infectious diseases, stress, hyperthermia, nutritional deficiencies, toxins, and autoimmune disorders (5, 6), has been notably attributed to testicular degeneration in dogs, with a reported prevalence of up to 57.6% (1, 3, 7). When some of these factors come into play, they can cause significant alterations in testicular structure, potentially leading to irreversible consequences for fertility. Testicular degeneration directly impacts the quality of the ejaculate, progressively impairing both sperm morphology and motility parameters (8). Therefore, current clinical approaches involve assessing the reproductive function of male dogs through sequential sperm analyses. When a decline in fertility is detected and treatment is initiated, reevaluation of the ejaculate after one full spermatogenic cycle is usually recommended. However, spermatogenesis in dogs spans approximately 62 days, with an additional 14 days for epididymal transit (9). Given the extended duration of this process, waiting for sperm production to improve may not be effective. Issues during this period can lead to a complete cessation of spermatogenesis (10). Therefore, timely intervention is crucial to avoid missing treatment opportunities. More objective assessments of testicular health can be achieved through diagnostic techniques such as histopathological and cytological examinations, following testicular biopsies and fine needle aspirations, respectively (11). However, due to their invasive nature and potential complications, both methods are rarely employed.

As an alternative, determining the concentration of anti-Müllerian hormone (AMH) in serum or plasma has recently emerged as a promising tool for the assessment of testicular function and integrity. Research conducted on mice has demonstrated that AMH is a reliable biomarker for detecting testicular damage secondary to aggressive chemotherapy (12, 13). Similarly, monitoring serum AMH levels has been recommended as a useful method for the early detection of testicular degeneration caused by toxic insults in stallions (14). AMH is a glycoprotein produced by Sertoli cells (15, 16). Therefore, the levels of AMH should be linked to Sertoli cell functionality, as it was scrutinized in bulls with heat-induced testicular degeneration (17). In dogs, results suggest that AMH secretion is increased not only in immature or tumorous Sertoli cells but also in degenerative Sertoli cells (18). Moreover, Sertoli cell degeneration may lead to decreased spermatogenic output, which is also marked by increased AMH production (19). Another study suggested that higher serum AMH concentrations were correlated with a lower percentage of morphologically normal spermatozoa (20).

Cryptorchidism is characterized by the failure of one or both testicles to descend into the scrotum (21). This condition results in the testicles being exposed to a different thermal environment compared to their normal scrotal location (8), which can lead to structural and functional changes (22–24) similar to those observed in non-cryptorchid individuals experiencing testicular degeneration and subsequent fertility drops (3).

In this study, cryptorchid dogs were used as models for impaired fertility and altered testicular histology. The aim was to assess the potential of AMH as a marker for testicular degeneration and impaired spermatogenesis. To achieve this, we investigated serum AMH levels in cryptorchid, intact, and castrated dogs, and analyzed AMH expression in both retained testicles and descended gonads. Finally, we examined the relationship between AMH levels, altered testicular histology, and impaired spermatogenesis.

## 2 Materials and methods

### 2.1 Sample collection

A total of 50 client-owned male dogs of various ages and breeds were recruited for the study. Twenty dogs with at least one retained testicle were classified into the CRYPTO group. Another 20 intact males with both testicles located inside the scrotum were assigned to the INTACT group. Additionally, 10 bilaterally gonadectomized dogs were included as negative controls, forming the CASTRATED group. All dogs underwent clinical examination, and no concerns were noted regarding their general or reproductive health. The cryptorchid patients were subjected to ultrasound examinations to confirm the position of the retained testicle prior to any proposed surgical intervention.

The composition of the three groups in terms of age, breed, unilateral or bilateral cryptorchid status, and the location of the retained testicle is detailed in Supplementary Table 1.

Blood samples were collected from all dogs participating in the serological study. The samples were drawn from the cephalic vein and stored in plain red-top collection tubes without additives (BD Vacutainer, Plymouth, UK). For CRYPTO and INTACT dogs, samples were taken before undergoing orchiectomy, and for dogs in the CASTRATED group, samples were collected at least 6 months post-castration. After allowing the blood to clot for 5 min at room temperature, the samples were centrifuged at 2,500×*g* for 10 min. The resulting serum was then stored at −80°C until further processing.

Ten dogs from each of the CRYPTO and INTACT groups underwent gonadectomy with owner consent. Both gonads were fixed in 10% neutral buffered formalin and subsequently embedded in paraffin blocks. For the unilateral cryptorchids, the contralateral descended gonad was available for analysis, with one exception. One unilateral cryptorchid had only the retained testicle available, as the scrotal one had been previously removed through surgery.

All sampling protocols underwent review and approval by the Ethical Committee of the Faculty of Veterinary Medicine, Bucharest (Approval Number: EA nr. 34-03/2024).

### 2.2 Evaluation of serum AMH levels

AMH serum levels were quantified using a validated three-step sandwich type immunoassay kit kit (Canine/Feline AL-116 ELISA, Ansh Labs, Webster, TX, USA), which has been previously used in dog-based studies (25, 26). Each coated well of the microplate was initially treated with 75 μL of Canine AMH Assay Buffer, in accordance with the manufacturer’s recommendations. Subsequently, after adding a volume of 25 μL of calibrators, controls, and serum samples, the plate underwent a 60-min incubation at room temperature on an orbital microplate shaker at 600 rpm. Following incubation, the wells were thoroughly washed, and 100 μL of Antibody-Biotin Conjugate was added to each well, followed by another incubation under the same conditions as per the manufacturer’s protocol. After additional washing steps, the wells were treated with Streptavidin-HRP conjugated enzyme for 30 min. The reaction was developed with a 10-min incubation using 100 μL of TMB chromogen solution and stopped with 100 μL of stopping solution. The plate absorbance was read at 450 nm, with background wavelength correction at 630 nm, using a PR 4100 Absorbance Microplate Reader (Bio-Rad Laboratories, Hercules, CA, USA). The intra-assay coefficient of variation was below 10%. Since all samples were processed simultaneously in a single batch, no inter-assay coefficient of variation was calculated. The detection limit of the kit was 0.015 ng/mL.

### 2.3 Tissue sample preparation

The previously stored paraffin blocks were sectioned repeatedly at 4 μm in order to create two different batches. The first one was stained with hematoxylin and eosin (HE) and directed to conventional microscopical evaluation, followed by histomorphometrical analysis and spermatogenesis assessment. The second batch was reserved for the immunohistochemical assessment.

Randomized sections from both descended (DES) gonads of the INTACT group were analyzed. All retained (RET) testicles from the admitted cryptorchid individuals were also analyzed. When available, the contralateral testicle (CONTRA) of unilateral cryptorchids was evaluated. A detailed description of sample availability for the histological and immunohistochemical assessments is presented in Table 1.

**Table 1:** Sample availability for histological and immunohistochemical assessments.

No	Breed	Age (months)	Left testicle		Right testicle	
			Location	Availability	Location	Availability
1	Mixed breed	48	Inguinal	Yes	Scrotal	Yes
2	Mixed breed	18	Scrotal	Yes	Inguinal	Yes
3	Mixed breed	24	Scrotal	No	Abdominal	Yes
4	Cocker Spagnel	14	Abdominal	Yes	Scrotal	Yes
5	Mixed breed	13	Inguinal	Yes	Scrotal	Yes
6	Mixed breed	14	Abdominal	Yes	Abdominal	Yes
7	Maltese	24	Abdominal	Yes	Abdominal	Yes
8	Pekingnese	18	Scrotal	Yes	Abdominal	Yes
9	Husky	12	Inguinal	Yes	Scrotal	Yes
10	Chihuahua	34	Inguinal	Yes	Scrotal	Yes
11	Mixed breed	26	Scrotal	Yes	Scrotal	Yes
12	Mixed breed	12	Scrotal	Yes	Scrotal	Yes
13	French Bulldog	12	Scrotal	Yes	Scrotal	Yes
14	Mixed breed	25	Scrotal	Yes	Scrotal	Yes
15	Mixed breed	12	Scrotal	Yes	Scrotal	Yes
16	German Shepherd	24	Scrotal	Yes	Scrotal	Yes
17	Mixed breed	12	Scrotal	Yes	Scrotal	Yes
18	Swiss Shepherd	26	Scrotal	Yes	Scrotal	Yes
19	Mixed breed	14	Scrotal	Yes	Scrotal	Yes
20	Mixed breed	12	Scrotal	Yes	Scrotal	Yes

#### 2.3.1 Immunohistochemical staining

The dedicated tissue sections were mounted on 3-aminopropyltriethoxysilane-coated slides. These slides were subjected to deparaffinization with xylene, followed by rehydration in decreasing concentrations of ethanol (100, 96, 50%) and 100% H_2_O. For antigen retrieval, the slides were placed in a box filled with distilled water and citrate buffer (pH 6.0), which was then placed inside a pressure cooker containing 600 mL of distilled water. The system (cooker + slides) was microwaved at 850 W for 12 min and then at 300 W for 10 min. After retrieval, the box containing the slides was cooled to room temperature for 20 min.

After a 5-min wash with PBS, 200 μL of H_2_O_2_ was added to each section for 5 min. The slides were then washed twice with PBS. The primary antibody used was anti-AMH C-terminal (anti-rabbit, Abcam, Cambridge, UK) at a 1:400 dilution (antibody diluent with background reduction, DAKO Agilent – S3022832, Santa Clara, CA, USA). The slides were incubated with the diluted primary antibody for 30 min, washed twice with PBS, and then exposed to 200 μL/slide of Envision Link rabbit (DAKO Agilent – K400311-2) for 30 min.

After this incubation, the slides were washed twice in PBS for 5 min and then treated with 200 μL/slide of 3,3-diaminobenzidine solution (DAB, DAKO Agilent – K346811-2) for a final 30-min incubation. Ultimately, the slides were washed in PBS again for 5 min, counterstained with hematoxylin, dehydrated, mounted with coverslips, and stored for further analysis.

### 2.4 Tissue sample analysis

Slides were assessed in a blinded manner, by one trained operator, using a Leica DM5500 B microscope (Leica Microsystems CMS GmbH, Wetzlar, Germany). Further image acquisition, digital processing and pixel analysis were performed using Leica Application Suite (LAS version 4.13, Wetzlar, Germany).

#### 2.4.1 General histology, spermatogenesis and histomorphometrical evaluation

Using the HE-stained slides, 20 sections of seminiferous tubules and the neighboring interstitial compartment were analyzed per sample. General histological traits specific to testicular degeneration were assessed, such as reduced seminiferous tubule diameter, decreased numbers of germinal cells, and interstitial fibrosis (27). For spermatogenesis evaluation, the presence of different cell types within the seminiferous tubules—spermatogonia, spermatocytes, spermatids, spermatozoa, and Sertoli cells—was assessed. Based on this assessment, a score from 1 to 10 was assigned to each of the 20 sections, following the Johnsen scale criteria adapted for use in dogs (28).

Moreover, the area of seminiferous tubules was determined using a methodology similar to the one previously used in stallions (29). Specifically, five round or nearly round tubules from the previously examined 20 sections were visualized at 400× magnification and measured across their major and minor axes. The seminiferous tubule area was calculated using the formula:


$$\mathrm{Seminiferous tubule area}=\pi \times \frac{\mathrm{major axis}}{2}\times \frac{\mathrm{minor axis}}{2}$$

#### 2.4.2 Tissue AMH expression quantification

Immunostaining quantification was performed through pixel analysis on five different randomly obtained images per sample, employing standardized color correction via white balance and a standardized threshold. The results were expressed as percentages of reactive areas relative to the total surface of two predefined Regions of Interest (ROIs). The ROIs were defined as follows: ROI^1^—the seminiferous tubule, including the entire intratubular area; and ROI^2^—the interstitial space between a minimum of two neighboring tubules. For both ROIs, the area was determined by tracking predefined margins and using the automated measuring tool specific to the LAS software.

### 2.5 Statistical analysis

The data were processed and subjected to statistical analysis using IBM SPSS version 18.0 for Windows (IBM Corp., Armonk, NY, USA). The normality of the variables was assessed using the Shapiro–Wilk test, with a significance level set at *α* = 0.05. Results were presented as median and interquartile range (IQR), outliers being identified and removed based on the IQR method. For comparisons across multiple groups, the Kruskal-Wallis test was applied to determine differences in the distribution of non-normally distributed data, followed by pairwise comparisons with significance values adjusted using the Bonferroni correction. For paired data without grouping variables, differences were evaluated using the Wilcoxon signed-rank test. Spearman’s rank correlation coefficient was employed to investigate potential associations within the dataset. Statistical significance was determined by considering *p*-values less than 0.05. The relation between the age and AMH variables was computed in all individuals included in the serological study and with the exclusion of the CASTRATED subjects. Afterwards, the remaining individuals were also split into young (age range 0–24 months) and adult (≥25 months).

**Figure 1:**
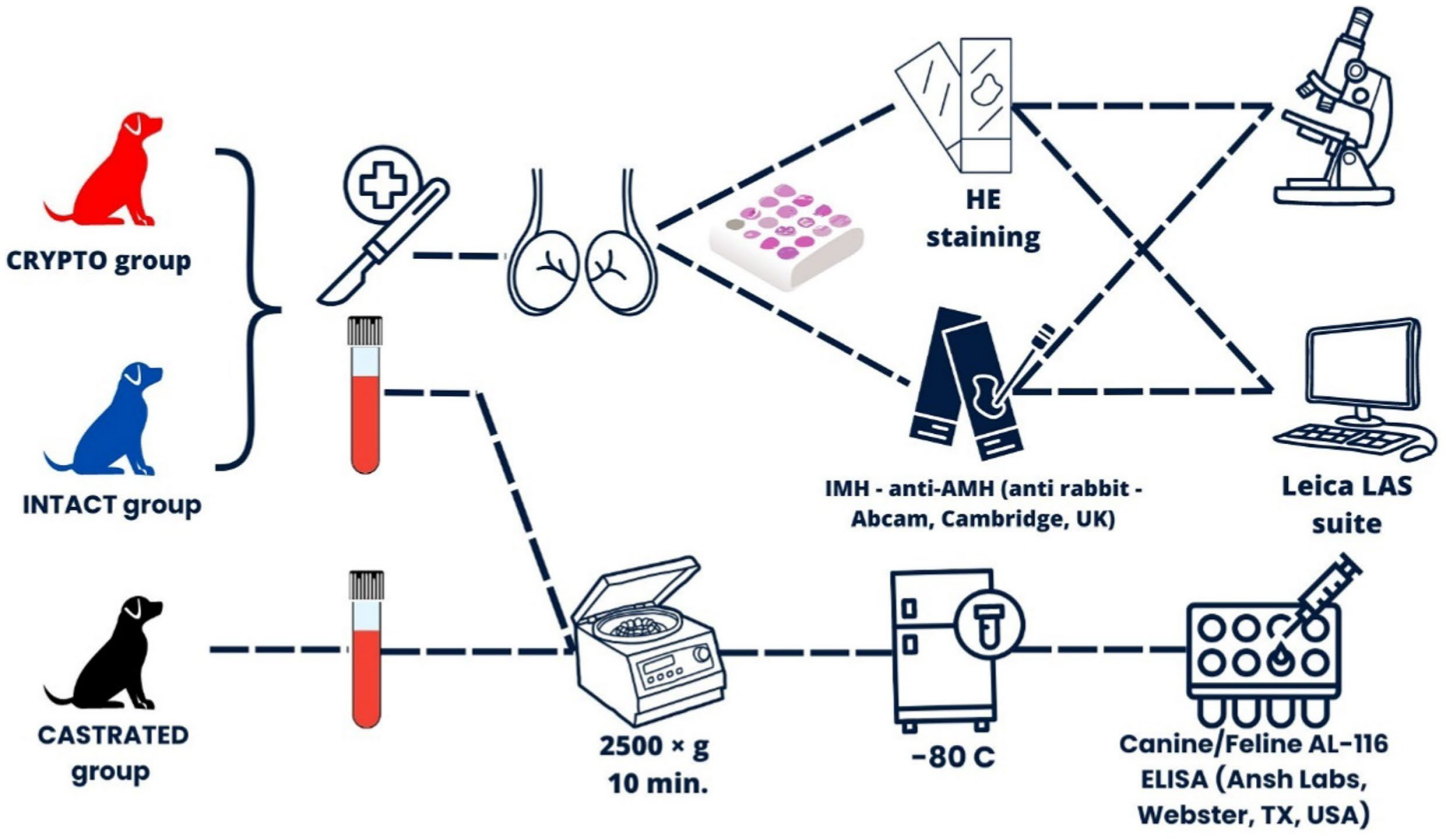
Experimental design overview illustrating the overall workflow, including the recruitment and classification of subjects into three main groups: CRYPTO (dogs with at least one retained testicle), INTACT (dogs with both testicles in the scrotum), and CASTRATED (dogs that had been castrated for more than 6 months prior to the study).

The experimental design is illustrated in Figure 1.

## 3 Results

### 3.1 AMH serum levels and age

**Figure 2:**
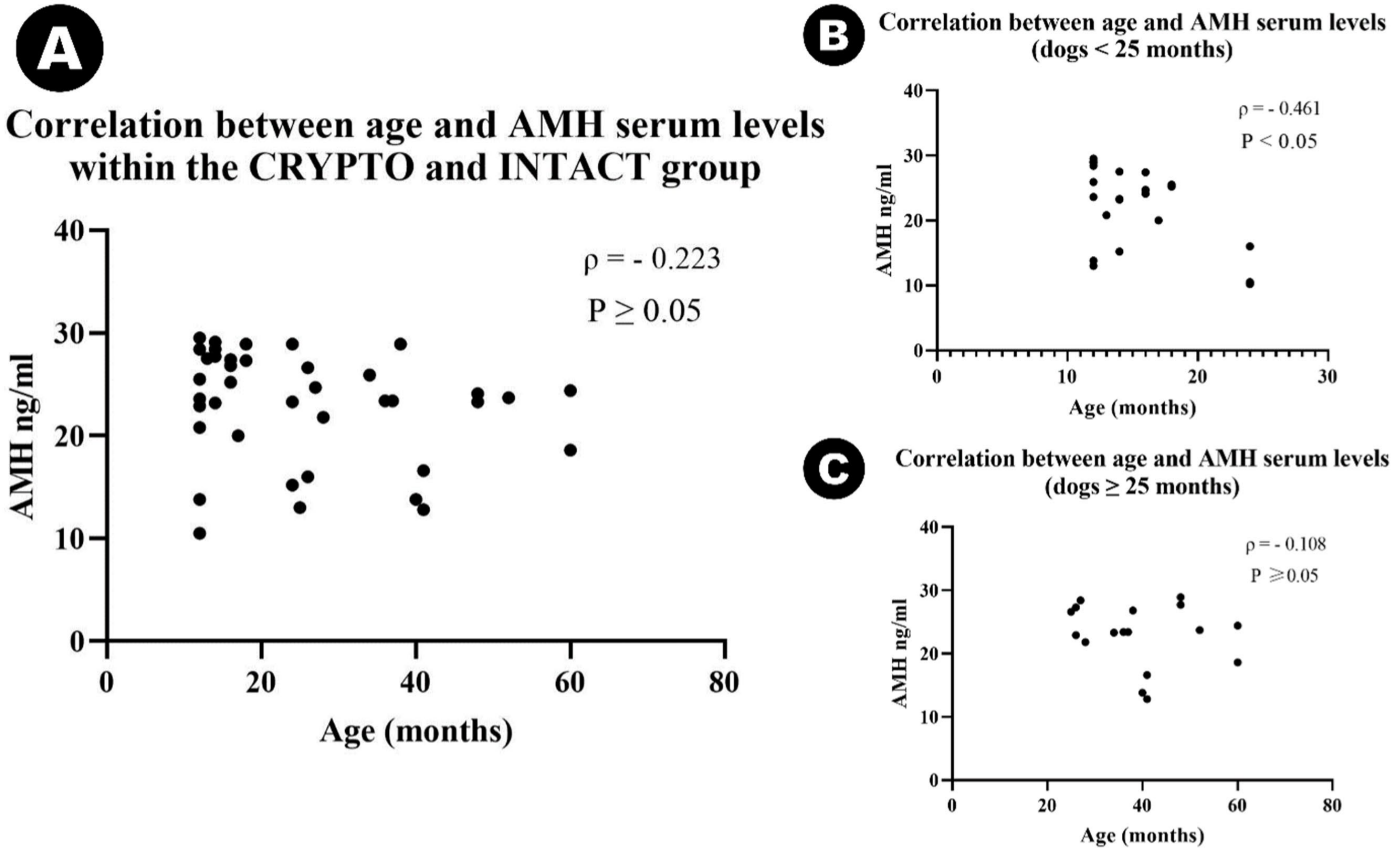
Violin plot representing median AMH serum concentrations among cryptorchid dogs (CRYPTO), intact males with both testicles in the scrotum (INTACT), and castrated males (CASTRATED) (A). Subcategories within the CRYPTO group (unilateral, bilateral, abdominal, and inguinal cryptorchids) are also depicted (B,C). Data are presented as median and quartiles (25th and 75th percentiles). The violin shapes illustrate the distribution of the data, the dashed lines indicate the medians, and the dotted lines represent the quartiles. Significance levels are indicated as *p* ≤ 0.05 (∗), *p* ≤ 0.01 (∗∗) and *p* ≤ 0.001 (∗∗∗).

**Figure 3:**
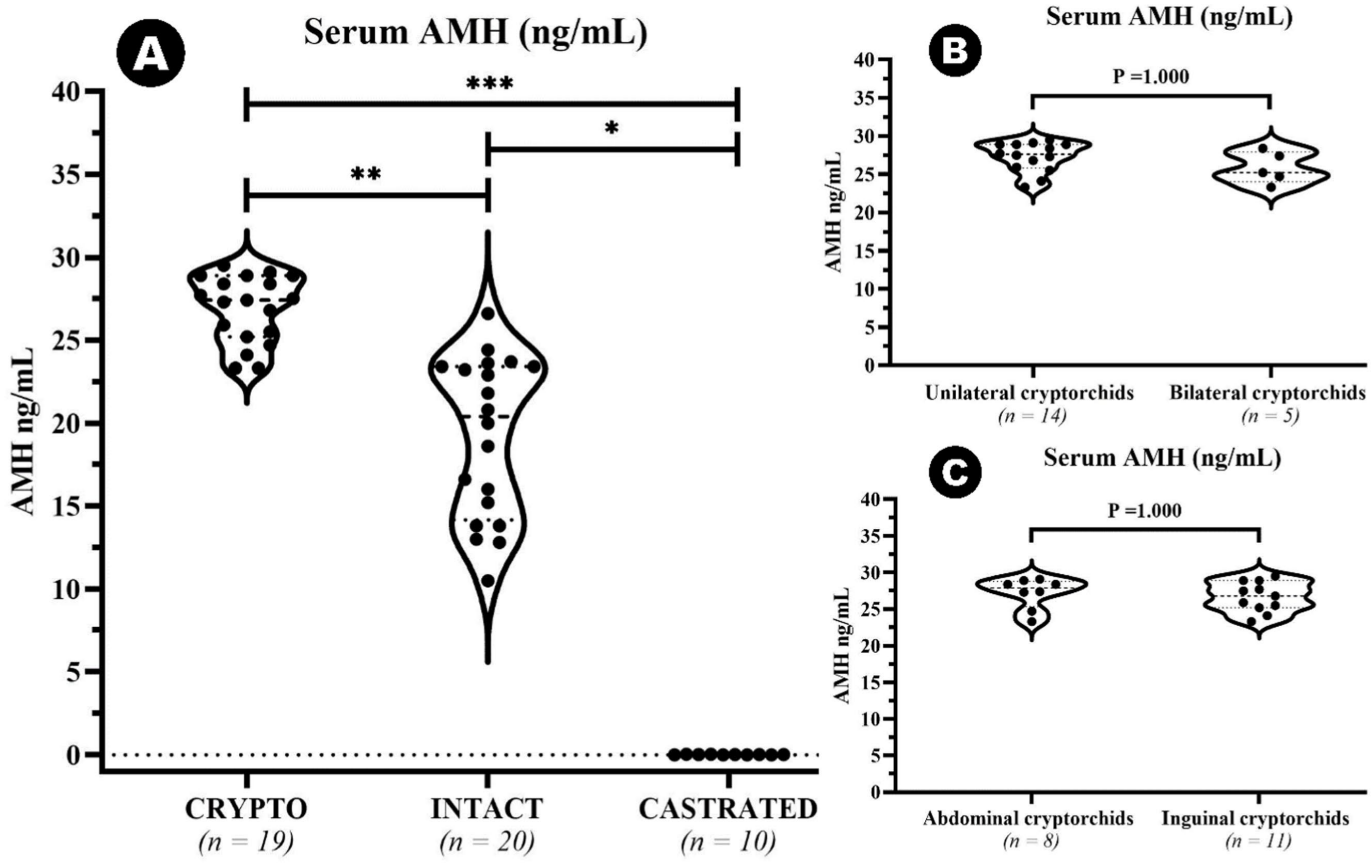
Scatter plots illustrating the correlation between age and AMH serum levels in different subgroups: (A) Scatter plot showing the correlation between age and serum AMH levels in cryptorchid dogs (CRYPTO) and intact males with both testicles in the scrotum (INTACT). (B) Scatter plot representing the correlation between age and AMH levels in CRYPTO and INTACT dogs aged 0–24 months. (C) Scatter plot representing the correlation between age and AMH levels in CRYPTO and INTACT dogs aged ≥25 months.

An outlier was identified in the CRYPTO group secondary to IQR calculations. The value of 10 ng/mL fell below the lower bound (15.65 ng/mL) and was therefore excluded from further analysis. Remaining data on serum AMH levels showed significant differences among specific groups (Figure 2). The CRYPTO group had significantly higher AMH levels (*n* = 19; median 27.4 ng/mL, IQR = 3.7) compared to both the INTACT (*n* = 20; median 20.4, IQR 9.25, *p* ≤ 0.01) and CASTRATED groups (*n* = 10; 0.01 ± 0.01 ng/mL *p* ≤ 0.001) (Figure 2A). The subjects from the CASTRATED group (*n* = 20) exhibited only serum AMH values below the described detection limit of the kit leading to a significant difference when compared to the INTACT AMH serum levels as well. However, no significant differences were detected between unilateral and bilateral cryptorchids (*p* = 1.000) (Figure 2B), nor between abdominal and inguinal cryptorchids (*p* = 1.000) (Figure 2C). After excluding the outlier in AMH values, the median age across all groups was 24 months (*n* = 49, IQR = 27). No statistically significant differences were observed in the age distribution among the CRYPTO, CASTRATED, and INTACT groups *p* ≥ 0.05. Notably, a negative correlation between age and AMH levels was detected across all groups (*ρ* = −0.415, *p* ≤ 0.01). However, when computing the analysis excluding the CASTRATED individuals—who typically have low AMH levels irrespective of age—the correlation between age and AMH values remained negative (*ρ* = −0.223) but was no longer statistically significant (*p* ≥ 0.05) (Figure 3A). Moreover, when the relationship between age and AMH serum levels was investigated within the separated age groups, a negative correlation was identified between the two variables in dogs younger than 24 months (*ρ* = −0.461, *p* < 0.05) (Figure 3B). Conversely, this correlation was not significant in the adult category (≥25 months) (*ρ* = −0.108, *p* ≥ 0.05) (Figure 3C).

### 3.2 Tissue analysis

#### 3.2.1 General histology

**Figure 4:**
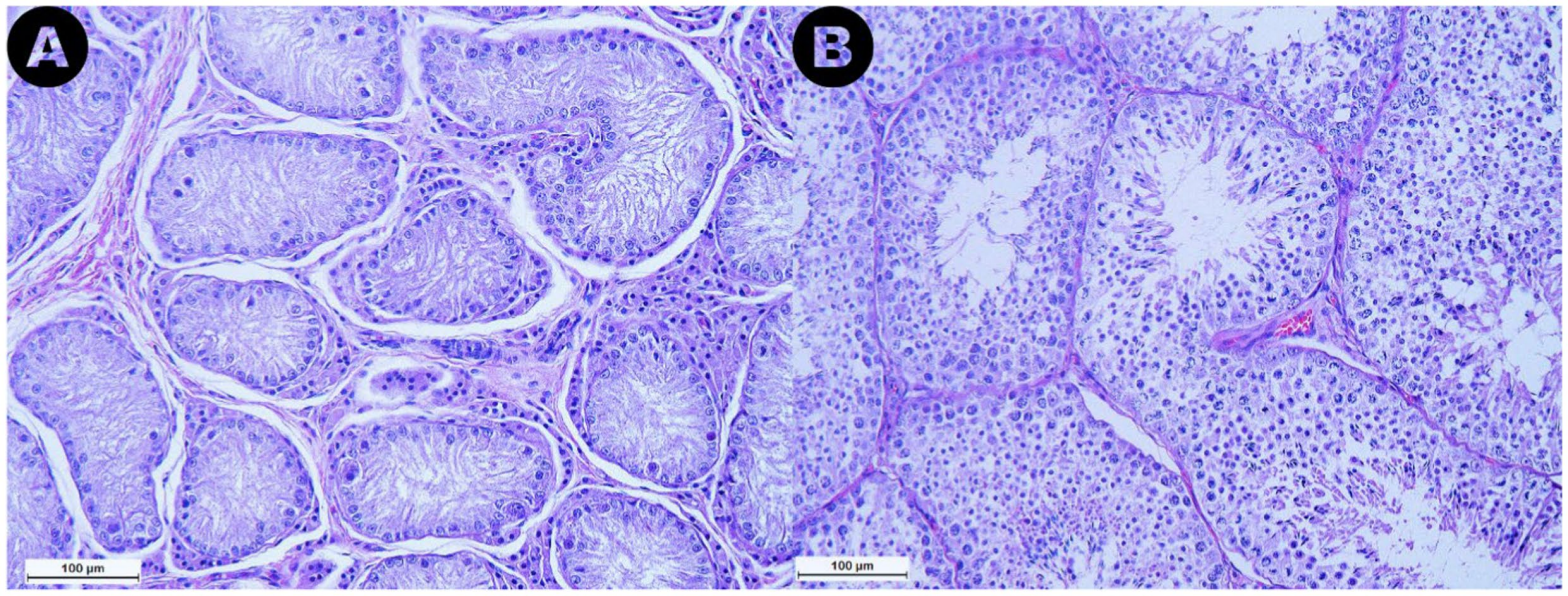
Microphotographs obtained from the retained (A) and the descended (B) testicles at a 200× magnification after Hematoxylin and Eosin staining. Note the marked reduction in size of the seminiferous tubules in the retained testicle compared to the descended one, along with the reduction or absence of the lumen observed in the retained gonad. Additionally, an increase in Leydig cell numbers and connective tissue in the interstitial space of the retained testicle can be noted.

The analyzed samples from the RET category exhibited seminiferous tubules with few germ cells, with 76.7% of the evaluated tubule sections being classified as Sertoli-cell-only tubules. Additionally, a marked reduction or absence of the lumen was noted (Figure 4A). Furthermore, seminiferous tubule degeneration was evident, characterized by a general loss of cellular detail. The basement membrane surrounding the tubules appeared thickened compared to samples from the CONTRA and DES groups. Moreover, in the interstitial space of the RET testicles, an increase in Leydig cell numbers was subjectively observed, accompanied by an increased amount of connective tissue (Figure 4A), when compared to the DES samples (Figure 4B).

#### 3.2.2 Seminiferous tubule areas

**Figure 5:**
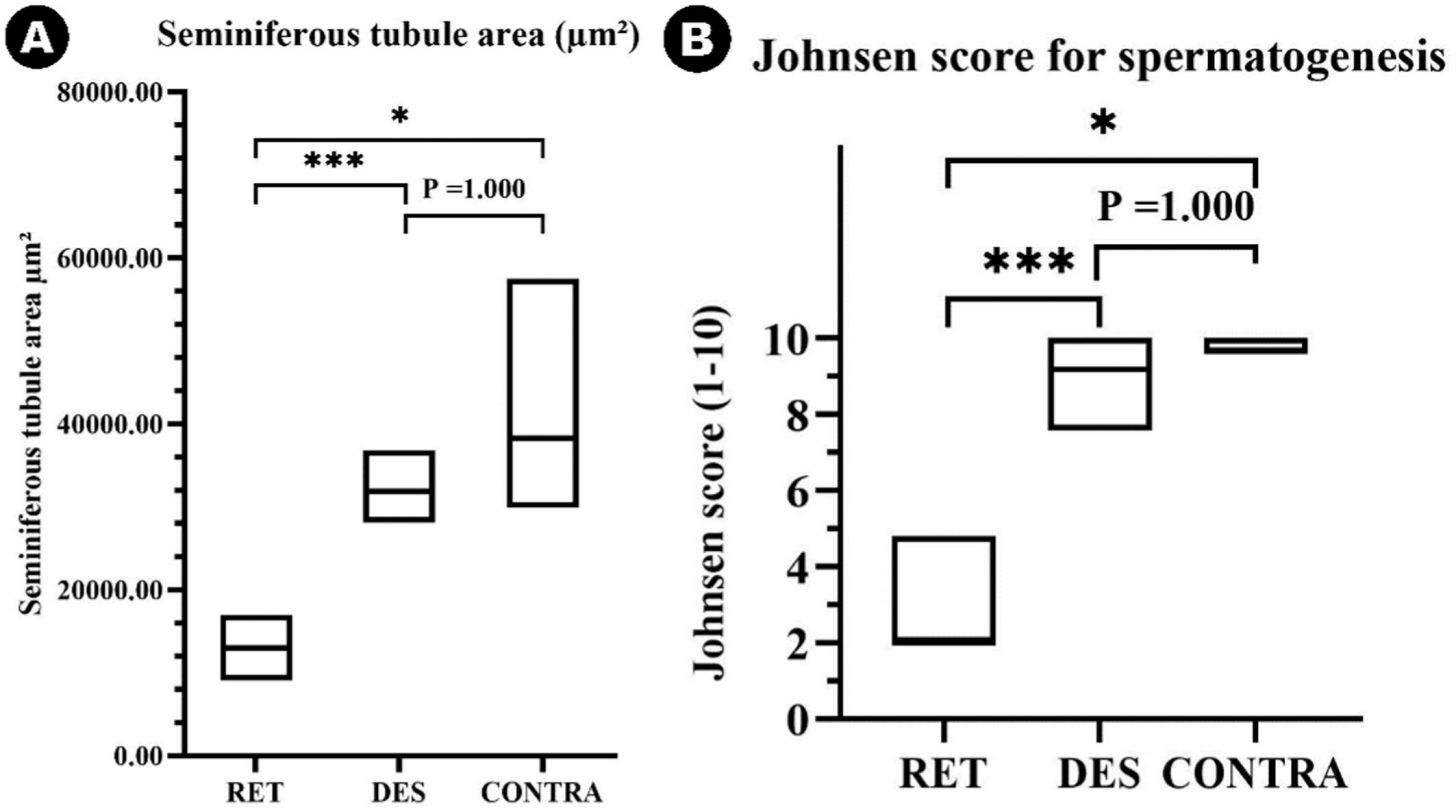
Floating bar graph depicting the seminiferous tubule areas (μm^2^) (A) and Johnsen score values (1–10) (B) across the retained testicles (RET), descended testicle samples from previously intact males with both testicles located in the scrotum (DES), and contralateral testicles from unilateral cryptorchids (CONTRA). Data is presented as floating bars (minimum to maximum). The line is equivalent to the median value of each group. Significance levels are indicated as *p* ≤ 0.05 (∗) and *p* ≤ 0.001 (∗∗∗).

Significantly lower tubule areas were found in the RET group (median 13,027.40 μm^2^, IQR = 3,764.41) when compared to the CONTRA (median 31,908.82 μm^2^, IQR = 7,061.17, *p* ≤ 0.05) and DES (median 38,339.93 μm^2^, IQR = 9,898.29, *p* ≤ 0.001) (Figure 5A). However, there was no significant difference in seminiferous tubule areas between the CONTRA and DES testicles (*p* = 1.000) (Figure 5A), nor when the distribution of the values was compared between the inguinally retained gonads and the abdominally located ones (*p* = 1.000). Interestingly, lower AMH serum values were specific to higher seminiferous tubule areas (*ρ* = −0.435, *p* ≤ 0.05).

#### 3.2.3 Johnsen score

**Figure 6:**
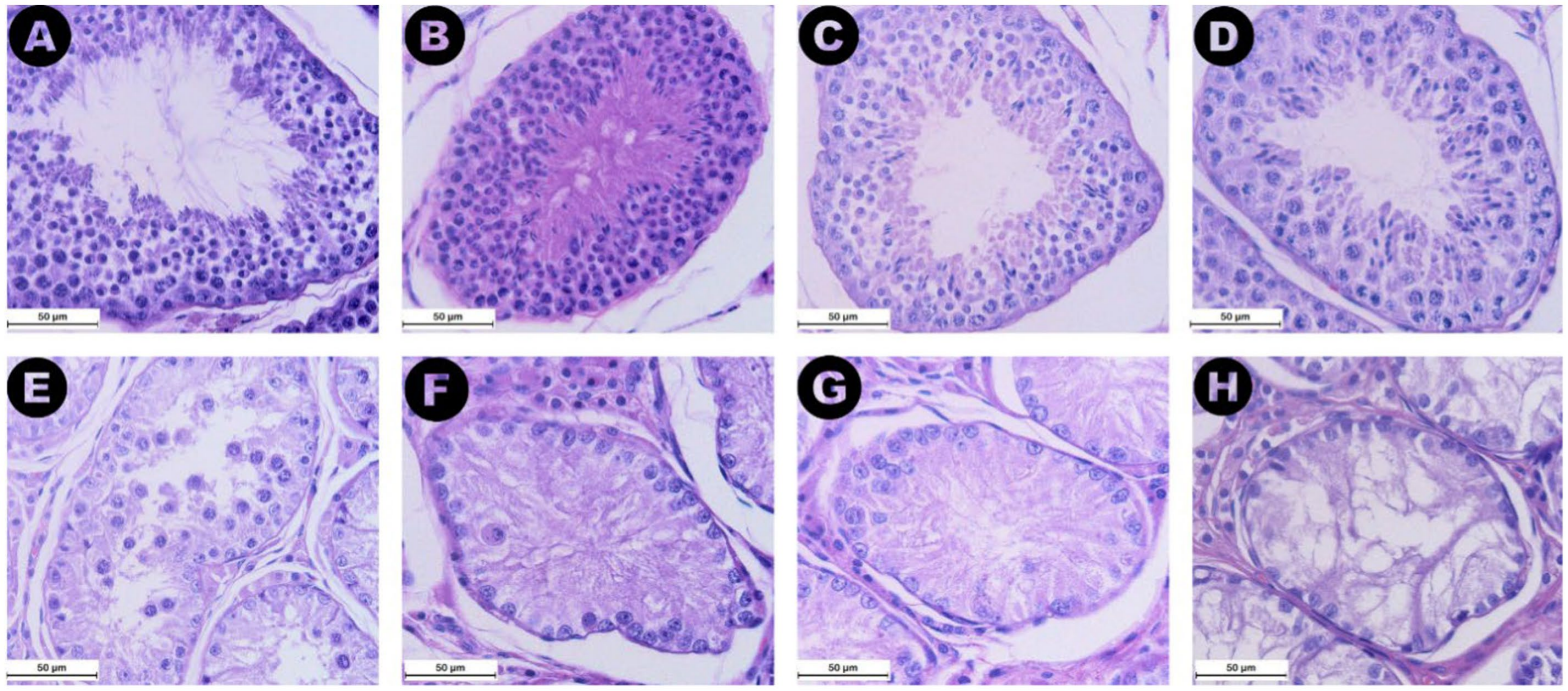
Images from (A) to (H) display 400× magnified microphotographs HE staining of seminiferous tubules graded according to the Johnsen score criteria defined by Merz et al. (28), with each image representing a different level of spermatogenic activity. (A) (Score 10) shows complete spermatogenesis with numerous spermatozoa. (B) (Score 9) depicts many spermatozoa (>10) but with disorganized epithelium and an obliterated lumen. (C) (Score 8) illustrates a low number of spermatozoa (<10). (D) (Score 7) features numerous spermatids (>10) but no spermatozoa. (E) (Score 5) displays several spermatocytes (>10) but no spermatozoa or spermatids. (F) (Score 4) shows a low number of spermatocytes (<5) without any spermatids or spermatozoa. (G) (Score 3) depicts seminiferous tubules with only spermatogonia present. (H) (Score 2) features tubules with only Sertoli cells and no germ cells. No images corresponding to scores of 6 or 1 were identified in the present groups.

All values of the Johnsen scale score have been assigned to the sections in the three categories except for scores 1 and 6 (Figure 6). The Johnsen score for the RET category (median 2, IQR = 0.5) was significantly lower than both the CONTRA (median 9.2, IQR = 0.8, *p* ≤ 0.05) and DES groups (median 9.7, IQR = 0.4, *p* ≤ 0.001) (Figure 5B). Additionally, higher scores were associated with lower AMH serum levels (*ρ* = −0.537, *p* ≤ 0.01) and bigger seminiferous tubules (*ρ* = 0.828, *p* ≤ 0.001). Similar to the seminiferous tubule areas, the comparison between the distribution of Johnsen scores assigned to the inguinally retained and abdominally retained gonads did not show significant differences (*p* = 1.000).

#### 3.2.4 Tissue AMH expression quantification

**Figure 7:**
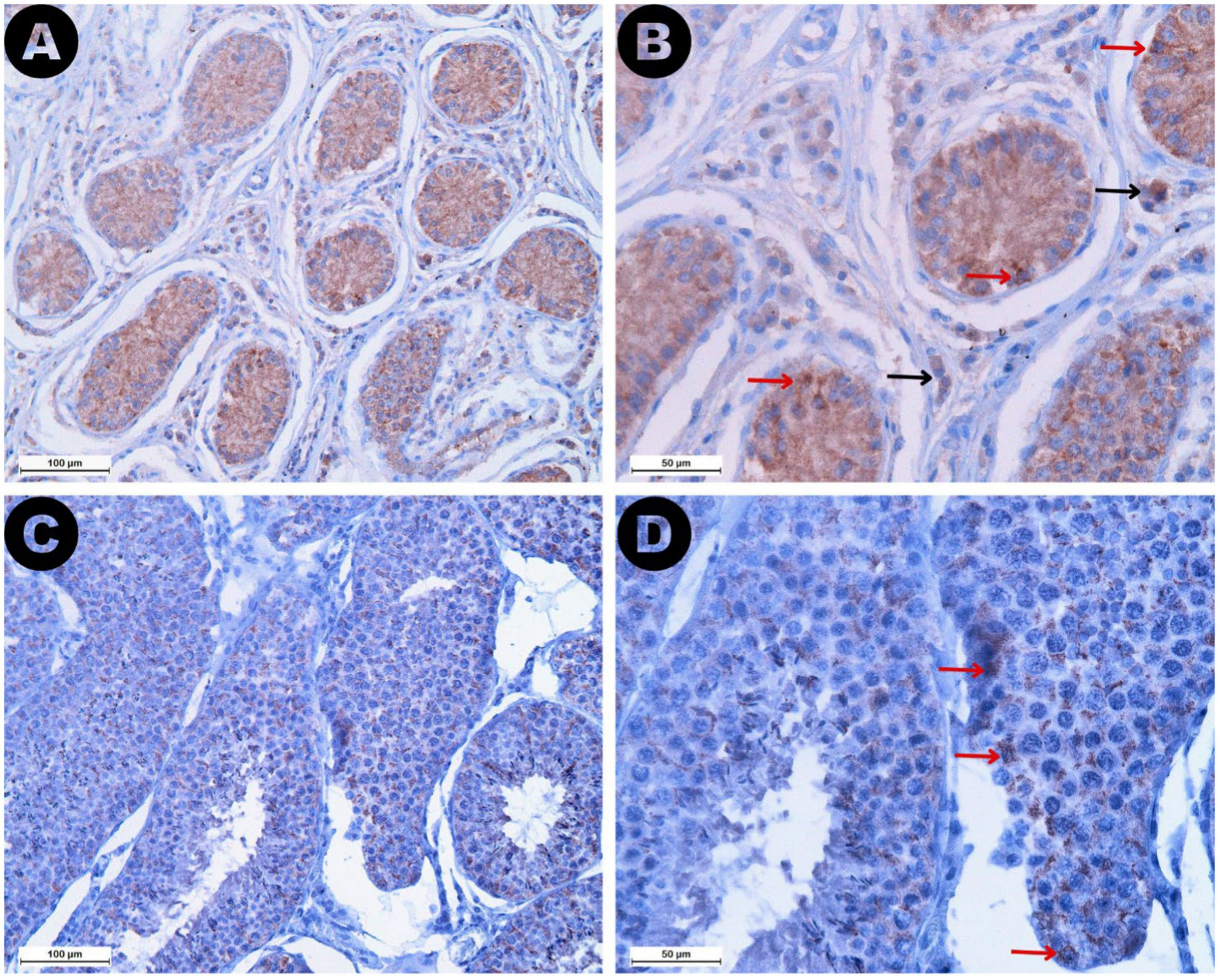
AMH immunostaining in both retained (A,B) and descended testicles (C,D) is depicted at different magnifications: 200× (A,C) and 400× (B,D). Note the higher AMH expression identified in the seminiferous tubules of the retained testicles compared to the descended gonads. Red arrows mark reactive Sertoli cells identified in both sample types, but lower intensity staining and fewer reactive cells were specific to the descended testicle samples. Black arrows show reactive Leydig cells identified exclusively in the interstitial space of the retained testicles.

The AMH expression was revealed as brown staining in both ROIs (Figure 7). Higher AMH expression was observed in the Sertoli cells of retained testicles, resulting in significantly higher expression levels in the seminiferous tubules of the RET group (median 32.42%, IQR = 15.11) compared to the CONTRA group (median 9.25%, IQR = 5.55, *p* ≤ 0.05) and the DES group (median 4.63%, IQR = 1.43, *p* ≤ 0.001). However, the difference in AMH expression between the CONTRA and DES groups was not significant (*p* = 0.444). The reactivity in the interstitial space ROI was lower than the intratubular expression across all samples (Z = −4.703, *p* < 0.001). Group-based comparisons showed that interstitial space reactivity values within the DES samples (median 0.05%, IQR = 0.08) was lower compared to the RET samples (median 2.36%, IQR = 3.24, *p* ≤ 0.001), but did not significantly differ from the CONTRA samples (median 0.12%, IQR = 0.15, *p* = 1.000). Moreover, intratubular and interstitial AMH expression levels did not substantially vary between inguinally retained testicles (*p* = 1.000) and abdominally retained gonads (*p* = 1.000).

Interestingly, serum AMH values were positively correlated with the tissue AMH expression in both the interstitial space (*ρ* = 0.494, *p* ≤ 0.01) and the seminiferous tubules (*ρ* = 0.610, *p* ≤ 0.001). Conversely, smaller seminiferous tubules were associated with higher AMH reactivity in both ROIs (seminiferous tubule: *ρ* = −0.774, *p* ≤ 0.001; interstitial space: *ρ* = −0.725, *p* ≤ 0.001). Similarly, Johnsen scores decreased as tissue AMH expression increased in the two analyzed compartments (seminiferous tubule: *ρ* = −0.756, *p* ≤ 0.001; interstitial space: *ρ* = −0.679, *p* ≤ 0.001). All correlations between the analyzed variables are briefly presented in Table 2.

**Table 2:** Spearman’s rank correlation matrix of the analyzed variables.

		Serum AMH	Seminiferous tubule area	AMH reactivity in ROI^1^^a^	AMH reactivity in ROI^2^^b^	Johnsen score
Serum AMH	Correlation coefficient	1.000	−0.435*	0.610**	0.494**	−0.537**
	*p*-value	–	0.018	<0.001	0.006	0.003
Seminiferous tubule area	Correlation coefficient	−0.435*	1.000	−0.774**	−0.725**	0.828**
	*p*-value	0.018	–	<0.001	<0.001	<0.001
AMH reactivity in ROI^1^^a^	Correlation coefficient	0.610**	−0.774**	1.000	0.831**	−0.756**
	*p*-value	<0.001	<0.001	–	<0.001	<0.001
AMH reactivity in ROI^2^^b^	Correlation coefficient	0.494**	−0.725**	0.831**	1.000	−0.679**
	*p*-value	0.006	<0.001	<0.001	–	<0.001
Johnsen score	Correlation coefficient	−0.537**	0.828**	−0.756**	−0.679**	1.000
	*p*-value	0.003	<0.001	<0.001	<0.001	–

This table displays the Spearman’s rank correlation coefficients and associated two-tailed *p*-values, highlighting the relationships among serum anti-Müllerian hormone (AMH) levels, seminiferous tubule areas, AMH reactivity in two defined regions of interest (ROIs), namely ROI^1^ and ROI^2^, and the Johnsen scores within the study cohort.

a ROI^1^ = region of interest defined as the area within the seminiferous tubule.

b ROI^2^ = region of interest defined as a limited area within the interstitial space between a minimum of two neighboring tubules.

*Correlation is significant at the 0.05 level (2-tailed).

**Correlation is significant at the 0.05 level (2-tailed).

## 4 Discussion

In this study, the potential of AMH as a biomarker for testicular degeneration and impaired spermatogenesis was assessed. Serum AMH levels and tissue AMH expression were evaluated in relation to histological findings specific to the degenerative processes and spermatogenic arrest identified in selected cryptorchid dogs.

Similar to other studies (26, 30), our results demonstrated the ability of serum AMH to identify the presence of functional testicular tissue, as evidenced by the significant differences observed between castrated and intact males. Moreover, the assay successfully differentiated cryptorchid individuals from those with scrotal-located gonads, which aligns with previous studies (30–32). However, in our study, it was unable to distinguish between unilateral and bilateral cryptorchidism or determine the location of the retained testicle. These findings suggest that AMH levels may be more closely related to the intensity of the degenerative processes than to whether one or both gonads are altered. This is further supported by the observation that, regardless of the location or the bilateral or unilateral cryptorchid state, AMH levels were higher in individuals with smaller seminiferous tubules and lower Johnsen scores.

Multiple human-based studies have consistently demonstrated a negative correlation between age and serum AMH levels, indicating a decline in AMH as age increases (33, 34). One particular study also reported variability in the rate of this decline among individuals, which was attributed to differences in the preservation of Sertoli cell output during aging (34). In our study, when castrated individuals were excluded from the analysis, this correlation between age and AMH levels was no longer supported. The relationship between these variables was evident only when the results were stratified by age group, suggesting that this association might be more relevant to younger individuals. Nevertheless, due to the limited sample size and uneven age distribution in the study, these findings should be interpreted with caution in dog populations, as they do not establish a definitive trend.

Interestingly, two studies in dogs revealed a correlation between increased AMH levels and a decline in sperm quality (19, 20). However, a relationship between age and AMH levels was observed in only one of these studies (19). This indicates that aging alone may not fully account for variations in AMH levels, and that these changes could be more closely related to structural and functional alterations in the testes that occur secondary to aging (28). Nonetheless, the relationship between age, AMH, and testicular degeneration remains unclear, as neither study (19, 20) explored the potential connection between degenerative changes in the testes and AMH levels within the context of sperm quality assessments.

Structural degenerative changes in canine cryptorchid testicles have been already described, including the reduction of seminiferous epithelium and tubule size along with cellular degeneration leading to arrested spermatogenesis (35, 36). Furthermore, these alterations were investigated together with AMH levels in cryptorchid stallions, revealing that smaller seminiferous tubules and lower Johnsen scores are associated with higher AMH serum levels (29), similar to our results. In cryptorchid patients, these structural and functional disruptions are explained by the effects of heat stress resulting from the different temperature regime to which the retained testicle is subjected. Heat stress may exert its effects by downregulating specific binding proteins, thereby interfering with the spermatogenetic process (37, 38). Moreover, elevated temperatures can lead to oxidative stress, which consequently results in germ cell degeneration and may impair Sertoli cell function (35, 39–41). The impact of heat stress on Sertoli cells has also been demonstrated in non-cryptorchid patients (17). A study involving bulls subjected to scrotal insulation revealed that heat-induced Sertoli cell dysfunction can lead to alterations in AMH levels, even in the absence of a cryptorchid state (17). This effect was explained by the capacity of heat stress to induce the reversion of Sertoli cells to an immature prepubertal phenotype (42), similar to cryptorchid testicles (43). In fact, this dematuration of Sertoli cells was considered the main reason for serum AMH increase when other factors were responsible for inducing acute testicular degeneration in stallions (14). The same approach was taken in mice and humans exposed to gonadotoxic treatments (12). The administration of doxorubicin and busulfan resulted in elevated serum AMH levels, which correlated with a significant reduction in seminiferous tubule size and a marked loss of meiotic germ cells, including spermatocytes, spermatids, and spermatozoa (12), in line with our findings in cryptorchids.

According to our results, AMH expression was higher in the seminiferous tubules of the retained testicles compared to the descended gonads, due to the more reactive Sertoli cells of the cryptorchid testicles. In other words, the tissue AMH expression intensified in the compressed tubules showing either few germ cells or Sertoli cells-only. These findings align with published data on cryptorchid stallions and tomcats in which AMH staining was negatively correlated with seminiferous tubule areas and Johnsen scores (29, 44). In dogs, higher AMH immunostaining in retained testicles was also explained by the presence of fewer germ cells in the seminiferous tubules, when compared to scrotal gonads (31). Therefore, AMH tissue expression appears to increase due to structural or functional changes within the testicle, associated with Sertoli cell dedifferentiation or dematuration, leading to elevated AMH serum levels (14, 45). Severely damaged mouse testicles showed increased AMH staining intensity, primarily identifying reactive Sertoli cells, and correlating with a higher degree of apoptosis (12). In testicles of infertile men, tubular atrophy was marked by the co-expression of AMH and CK-18 in the Sertoli cells (45). This expression pattern was specific for the tubules showing spermatogenic arrest at the spermatogonia stage, as well as in tubules displaying Sertoli cells only (45), similar our Johnsen score 2 and 3 tubules.

The AMH receptor was initially thought to be located exclusively on the membrane of Sertoli cells, but subsequent research has also identified its presence on Leydig cells (46). Moreover, the interaction between AMH and the receptors located on Leydig cells may have the potential to inhibit testosterone production (46). Interestingly, in retained testicle samples a higher reactivity was noted in the interstitial space in comparison to the same compartment of the descended gonads, suggesting a higher interaction between the above-mentioned receptors and the AMH. Therefore, we may consider that AMH could also reflect the steroidogenic capacity of testicular tissue, which may be altered by degenerative insults such as those seen in stallions with thermally induced testicular degeneration (47).

The contralateral descended testicles of unilateral cryptorchids did not significantly differ from the descended testicles of intact individuals in any of the analyzed aspects. These findings are consistent with previous observations in dogs, where similar comparisons were made across three different age categories, examining various developmental stages of cryptorchids (35). In this light and considering that serum AMH was unable to differentiate between unilateral and bilateral cryptorchids, we can hypothesize that AMH might not be effective in indicating whether one or both testicles are undergoing testicular degeneration. However, it could still be a valuable marker for raising awareness when structural changes begin to develop in at least one of the gonads.

In spite of the valuable aspects of this study, there are some limitations to consider. One limitation is the lack of ejaculate collection from the dogs prior to castration, which would have provided important insights into sperm functionality and morphology at the time of blood sampling. Another limitation is the use of cryptorchid dogs as models for testicular degeneration, along with healthy controls that lacked a complete reproductive history. Future studies could focus on dogs suffering from various degrees of degeneration due to other causes, such as mechanical insults or neoplasia, to deepen the understanding of testicular degeneration mechanisms. Additionally, including only dogs with proven fertility as healthy controls could offer more robust insights for future research.

In conclusion, this study supports the use of AMH as a biomarker for testicular degeneration, as demonstrated in cryptorchid dogs. This condition exhibits structural and functional changes similar to those seen in descended testicles affected by agents such as heat stress or gonadotoxic compounds, which also induce testicular degeneration. Elevated serum AMH levels and increased AMH tissue expression in retained testicles were closely correlated with structural damage and impaired spermatogenesis, indicating that AMH reflects significant alterations within testicular tissue. While serum AMH proves to be a promising minim-invasive tool for the early detection of testicular dysfunction, its current limitations in assessing the severity of degeneration highlight the need for further research and complementary diagnostic methods to enhance the evaluation of testicular health and fertility in male dogs.

## Data Availability

The original contributions presented in the study are included in the article/Supplementary material, further inquiries can be directed to the corresponding author.
