# Supplementary material for: Anti-Müllerian hormone as a diagnostic marker for testicular degeneration in dogs: insights from cryptorchid models
Source: Front Vet Sci. 2024 Oct 7;11:1481248. doi: 10.3389/fvets.2024.1481248 (PMC11493918; doi:10.3389/fvets.2024.1481248)
Supplement: Supplementary file 1 [file Data_Sheet_1.docx]

Supplementary Material

# Supplementary table

| No | Breed | Age (months) | Testicular condition | Testicle location |
| --- | --- | --- | --- | --- |
| CRYPTO group | | | | |
| 1 | Chihuahua | 38 | Unilateral cryptorchid | Inguinal * |
| 2 | Maltese dog | 12 | Unilateral cryptorchid | Abdominal * |
| 3 | American Staffordshire | 12 | Unilateral cryptorchid | Abdominal * |
| 4 | Cocker Spaniel | 14 | Unilateral cryptorchid | Abdominal * |
| 5 | Siberian Husky | 12 | Unilateral cryptorchid | Inguinal * |
| 6 | Mixed breed | 14 | Bilateral cryptorchid | Abdominal |
| 7 | Maltese dog | 24 | Bilateral cryptorchid | Abdominal |
| 8 | Pekingese | 18 | Unilateral cryptorchid | Abdominal * |
| 9 | Mixed breed | 13 | Unilateral cryptorchid | Inguinal * |
| 10 | Chihuahua | 34 | Unilateral cryptorchid | Inguinal * |
| 11 | Golden Retriever | 16 | Unilateral cryptorchid | Inguinal * |
| 12 | Mixed breed | 16 | Bilateral cryptorchid | Inguinal |
| 13 | Pomeranian | 12 | Unilateral cryptorchid | Inguinal * |
| 14 | Pomeranian | 14 | Unilateral cryptorchid | Inguinal * |
| 15 | German Shepherd | 16 | Bilateral cryptorchid | Abdominal |
| 16 | Miniature Pinscher | 48 | Unilateral cryptorchid | Inguinal * |
| 17 | Mixed breed | 27 | Bilateral cryptorchid | Abdominal |
| 18 | Mixed breed | 48 | Unilateral cryptorchid | Inguinal * |
| 19 | Mixed breed | 18 | Unilateral cryptorchid | Inguinal * |
| 20 | Mixed breed | 24 | Unilateral cryptorchid | Abdominal ** |
| INTACT Group | | | | |
| 21 | Mixed breed | 26 | Intact | Scrotal |
| 22 | Mixed breed | 12 | Intact | Scrotal |
| 23 | French Bulldog | 12 | Intact | Scrotal |
| 24 | Mixed breed | 25 | Intact | Scrotal |
| 25 | Mixed breed | 12 | Intact | Scrotal |
| 26 | German Shepherd | 24 | Intact | Scrotal |
| 27 | Mixed breed | 12 | Intact | Scrotal |
| 28 | Swiss Shepherd | 26 | Intact | Scrotal |
| 29 | Mixed breed | 14 | Intact | Scrotal |
| 30 | Mixed breed | 12 | Intact | Scrotal |
| 31 | Golden Retriever | 40 | Intact | Scrotal |
| 32 | Golden Retriever | 52 | Intact | Scrotal |
| 33 | Chinese Crested Dog | 60 | Intact | Scrotal |
| 34 | Golden Retriever | 41 | Intact | Scrotal |
| 35 | Golden Retriever | 17 | Intact | Scrotal |
| 36 | Bullmastiff | 37 | Intact | Scrotal |
| 37 | Cane Corso | 60 | Intact | Scrotal |
| 38 | Siberian Husky | 28 | Intact | Scrotal |
| 39 | Siberian Husky | 41 | Intact | Scrotal |
| 40 | Siberian Husky | 36 | Intact | Scrotal |
| CASTRATED group | | | |  |
| 41 | Mixed Breed | 74 | Castrated |  |
| 42 | Mixed Breed | 48 | Castrated |  |
| 43 | Mixed Breed | 24 | Castrated |  |
| 44 | Mixed Breed | 72 | Castrated |  |
| 45 | Mixed Breed | 72 | Castrated |  |
| 46 | Mixed Breed | 24 | Castrated |  |
| 47 | Mixed Breed | 26 | Castrated |  |
| 48 | German Shepherd | 18 | Castrated |  |
| 49 | Mixed Breed | 24 | Castrated |  |
| 50 | Mixed Breed | 84 | Castrated |  |

**Supplementary Table 1.** Group composition by age, breed, testicular condition and location. * The location of the retained testicle is described, while the contralateral testicle is located in the scrotum. **Only the retained testicle was present (hemicastrated patient).
